# Psychosocial Impact of Epilepsy in Older Adults

**DOI:** 10.3390/healthcare3041271

**Published:** 2015-12-18

**Authors:** Rinu Manacheril, Urooba Faheem, David Labiner, Kendra Drake, Jenny Chong

**Affiliations:** Department of Neurology, University of Arizona, Tucson, AZ 85724, USA; E-Mails: rinugood@gmail.com (R.M.); urofah@gmail.com (U.F.); labinerd@email.arizona.edu (D.L.); kdrake@neurology.arizona.edu (K.D.)

**Keywords:** psychosocial impact, epilepsy, attitude, new onset epilepsy, elderly

## Abstract

*Objective*: The purpose of our study was to describe the quality of life of older adults with seizures or epilepsy and compare its psychosocial impact between those who were new diagnosed and those diagnosed before the age of 65. *Methods*: In-depth face to face interviews with open ended questions were conducted with two participant groups: Incident group: 42 older adults (>65 years) with new onset or newly diagnosed after age of 65; and Prevalent group: 15 older adults (>65 years) diagnosed before age of 65. Interviews were reviewed and coded using a list of themes and results were compared between the two groups. Eight topics were selected from the participants’ responses to questions about the psychosocial impact of epilepsy and seizures. The topics were then analyzed and compared between the two groups. *Results*: The topics analyzed were: Emotional and physical impact, significant life changes, co-morbidities, information gathering, stigma, AED side effects, changes in relationships and attitude toward diagnosis. *Conclusion*: We concluded that the age at onset and duration does seem to have a negative correlation with health related quality of life. However, the perceived health status of older adults with chronic epilepsy was significantly better and reflected in their more positive approach to the diagnosis of seizures or epilepsy probably because they have had a longer opportunity to learn to cope with their diagnosis.

## 1. Introduction

Epilepsy affects over 3 million people in the United States, with approximately 150,000 new cases each year. The incidence of epilepsy is bimodal being higher in the young and the old. The incidence is greatest among older adults (>55 years) primarily because some age related co-morbidities are epilepsy risk factors [1]. Epilepsy in older adults presents with its own set of unique challenges. Neurodegenerative, cerebrovascular, neoplastic and psychiatric co-morbidities often complicate the clinical picture among this population with epilepsy [2]. In addition, epilepsy interferes with issues vital to older adults such as being an active member of society, maintaining physical and mental capacities, and retaining autonomy, all of which are associated with successful aging [3,4].

Quality of life is determined by the physical, mental and social health of the individual. This includes psychological health, as well as the ability to maintain social roles, responsibilities and relationships [5]. Older adults with epilepsy were reported to have poorer quality of life compared to the general population, with significantly worse neuropsychological health, cognitive impairment and social isolation with stigma further compounding the problem [5,6]. Some research suggested that quality of life for adults with epilepsy varies with age of the patient, frequency of seizures, age of onset and duration of epilepsy and that older adults were better able to cope with epilepsy compared to middle-aged adults [3,4,7].

In contrast, another study disputed that finding and reported instead that quality of life is affected by seizure-related variables, co-morbidities and tolerability of anti-epileptic drugs (AEDs) [8].

The aim of our study was to describe the quality of life of seniors with epilepsy, comparing between those who were newly diagnosed with seizures or epilepsy and those who had been diagnosed earlier in life, specifically before age 65. In particular, this study assessed the contributions of age of onset, co-morbidities, tolerability of AED’s and other psychosocial factors on the quality of life of seniors with seizures. To that extent, the study was aimed at providing in-depth knowledge of the factors that contribute to health related quality of life in older adults with epilepsy. A recent European study that included patients with one of nine neuropsychiatric disorders (dementia, depression, epilepsy, migraine, multiple sclerosis, Parkinson’s disease, schizophrenia, stroke and substance dependence) suggested that regardless of clinical diagnoses or its severity, rehabilitation for emotional, sleep and work related problems should be provided for all neuropsychiatric conditions, one of which is epilepsy [9]. Understanding the psychosocial impact of epilepsy on older adults is crucial to healthcare professionals in identifying areas of intervention and improve their patients’ overall well-being.

## 2. Methods

In this multicenter, descriptive analysis, a convenient sample of 57 participants was obtained. Participants were seniors (aged 65 or older) who had experienced a seizure or were diagnosed with epilepsy and live in Southern Arizona. They were recruited between 2010 and 2013 for a study of the incidence of epilepsy among seniors as well as to describe the impact on seniors and their family following a diagnosis of a seizures or epilepsy. Inclusion criteria was based on: (a) age of discovery of epilepsy diagnosis being before or after 65 years; (b) residential domain being Arizona; and (c) current treatment with AED. Individuals were recruited through numerous avenues including media advertisements (magazines, local newspapers), flyers in clinics, provider referrals, and through public education sessions. Potential participants were sent a letter inviting them to participate in the hour-long in-depth interview, together with a consent form to review. They were then contacted by telephone and an appointment was made if the participant was interested in taking part. Participants were interviewed within a month of recruitment after obtaining informed consent. They were interviewed every six months if they were newly diagnosed, or just once, if they had a pre-existing diagnosis of seizures or epilepsy, made prior to 2010. The interview was designed to have the patient describe the period when they were first diagnosed, and their current living situation. The regular interviews were conducted over the telephone to enquire about whether there were changes in living arrangements, quality of life (adapted from the Behavioral Risk Factor Surveillance Survey), anxiety (GAD assessment), and depression (PHQ9 assessment). Topics for the in-depth interviews included the impact of being diagnosed with epilepsy or seizure, social relations, coping with seizures, treatment, and resources sought. The complete interview guide can be found elsewhere [10]. Additional contextual information offered by the participant and/or family member (including spouse or partner) was documented.

The recordings of these in-depth interviews were transcribed and checked for accuracy by a second transcriber. The transcripts were analyzed by three reviewers; each noted the responses to the questions posed and organizing them into themes. The reviewers then discussed the themes to ensure consistency of coding. Once a consensus was reached, each interview was reviewed again to ensure that the interviews had been assigned the appropriate codes [10]. In addition to the regular interviews, two complementary strategies were used to gather additional data—in depth interviews with some of the participants, and focus groups with the participants’ family member or spouse/partner. The in depth interview was conducted face-to-face, using open-ended questions to elicit more contextual information. Topics for the in-depth interviews include the impact of being diagnosed with epilepsy or seizure, social relations, coping with the diagnosis, treatment, and resources sought. Similar topics were also used in the focus groups with family members. Data collected was analyzed by Dr. Chong using SPSS (Statistical Package for Social Sciences) Version 20.0. This study was approved by The University of Arizona’s Institutional Review Board (IRB00000291).

### Coding for the In-Depth Interview

Interviews (all English) were recorded and transcribed. Initially, two reviewers read the transcriptions independently and met to agree upon a list of themes. Next, at least three separate reviewers reviewed each transcripts and coded the interviews using the list of themes. The coding of each interview was done at least twice, with pairs of reviewers. One of the reviewers was always constant (JC). Information from the regular interviews and by the family member during focus groups were also included if the content was directly pertinent to the themes being coded.

## 3. Results

In our sample, all participants were over the age of 65 years who had new onset seizures, newly diagnosed with seizure disorder/epilepsy or who have been diagnosed earlier in life. The Incident group consisted of 42 participants with new onset seizures or were newly diagnosed after the age of 65, while the Prevalent group consisted of 15 older adults over the age of 65 with seizures diagnosed before the age of 65. Table 1 describes the participant demographic, seizure, and mental health characteristics at the time of the interviews.

**Table 1:** Participant characteristics.

Characteristics	Prevalent Group N = 15		Incident Group N = 42	
	Mean	Std. Dev.	Mean	Std. Dev.
Current age (year)	70.7	3.8	76.2	6.7
Age of Diagnosis (year)	39.9	19.9	73.2	10.8
	**Frequency**	**Percent**	**Frequency**	**Percent**
**Sex**				
Female	9	60.0	27	64.3
Male	6	40.0	15	35.7
**Income**				
Under $15,000	4	26.7	2	4.8
$15,000–$34,999	0		10	26.2
$35,000–$49,000	4	26.7	10	23.8
$50,000–$99,999	3	20.0	14	33.3
$100,000 and over	1	6.7	2	4.8
**Marital status**				
Married	8	53.3	26	61.9
Divorced	2	13.3	3	7.1
Widowed	1	6.7	11	26.2
Never been married	4	26.7	2	4.8
**Living arrangements**				
Alone	2	13.3	9	21.4
With spouse	9	60.0	25	59.5
With children	1	6.7	2	4.8
With other family	3	20.0	3	7.1
With people other than family	0	0	1	2.4
Other	0	0	2	4.8
**Education**				
Elementary	1	6.7	1	2.4
Some high school	0	0	1	2.4
High School Graduate	2	13.3	14	33.3
Some College/ Tech School	5	33.3	9	21.4
College	7	46.7	17	40.5
**Age of seizure onset (year)**				
0–9	2	13.3		
10–20	2	13.3		
21–44	4	20.0	1	2.4
45–64	8	53.3	6	14.3
65–74			13	31.0
75–84			19	45.2
85+			3	7.1
**Receiving depression and/or anxiety treatment**				
Yes	7	46.7	26	61.9
No	8	53.3	13	31.0
**Still on seizure medication**				
Yes	14	93.3	40	95.2
No	1	6.7	2	4.8
**Six months seizure free**				
Yes	7	46.7	34	81
No	8	53.3	8	19

Interviewees were asked directly how the seizures had affected their quality of life. Several themes emerged, ranging from either no impact or a refusal to let their quality of life be affected, to others that include feeling their social life had been restricted, affected by medication side effects, losing independence and being unable to accomplish or do as much as they used to or wanted to.

Responses to other questions were analyzed to determine how participants describe the various aspects of psychosocial impact seizures had. Upon review of all the interviews, there were several themes that were common and resonated within this participant population. These themes were grouped under the eight topics described below to collectively describe quality of life: Emotional and physical impact, significant life changes, co-morbidities, information gathering, stigma, AED side effects, changes in relationships and attitude toward diagnosis. Responses for each topic was compared between the two groups.

### 3.1. Emotional and Physical Impact

Participants from both groups reported emotional and physical symptoms that had affected their quality of life.

More than half the Incident (59.5%) and the Prevalent group (78.5%) reported emotional symptoms of depression and anxiety as a result of living with seizures or epilepsy, with most of them receiving mental health treatments. At the time of diagnosis, several who were diagnosed as young adults (Prevalent group) reported having negative emotions (*i.e.*, upset, devastated, angry) because they perceived that their life would have to change. One member of the Prevalent group said, “In the beginning, it was terribly, terribly depressing, shocking…I didn’t tell anyone for years which made my working life a little harder but I really didn’t want to tell people about it”. A member of the Incident group said, “It is depressing…not as active as I used to be or travel as we planned”.

Physical symptoms were also described by about half the participants in both groups reported more fatigue, drowsiness, trouble with memory and concentration. One participant from the Incident group noted, “I feel somewhat drugged all day…it slows you down and makes it impossible to do the things you like”. A participant from the Prevalent group stated, “I feel like I need more than 8 hours sleep now…I don’t schedule much in the morning if I can help it because I am now slow first thing in the morning”. Participants from both groups reported physical trauma sustained during episodes of seizures resulting in limitations to their physical activities. One participant from the Prevalent group said, “I wound up having a seizure and an intern grabbed my shoulder and lifted me…he dislocated my shoulder and then broke one of my bones…I have three screws in my shoulder now”. Not everyone attributed their somatic symptoms to seizures; some in the Incident group suggested that their symptoms were age-related, while others mentioned that pre-existing or other co-morbidities were more limiting.

### 3.2. Significant Life Changes

Due to the unpredictable nature of seizures, half of the Incident (50.0%) and more of the Prevalent (57.1%) group reported making lifestyle changes. These changes included measures to minimize seizure triggers, ensure adequate control of risk factors that might lower seizure threshold and exercising caution when involved in activities that carry risk. Initially, several of the participants from the Prevalent group reported that they did not allow the diagnosis to define them. They initially tried to continue on with their life, unaltered. However, they made adjustments later on in their life. For example, one refused to accept his diagnosis or take precautions earlier in his life, resulting in the loss of his driver’s. Since then, as a result of accidents, he had become more careful, making his environment safer for himself. Another individual worked for 32 more years following the diagnosis and remained active outdoors (with hunting, fishing and scuba diving) before retiring at age 65. The spouse of one participant in the Prevalent group noted: “We don’t have any glass doors on showers…he tries to remember not to go into the pool himself…he is not supposed to iron”.

Seizures and seizure management were responsible for about a third of the participants from the Incident (33.3%) and the Prevalent group (35.7%) reporting significant life changes. Events that constituted major life changes included not driving (at least temporarily), loss of independence secondary to requiring assistance to carry out activities of daily living, early disability or a change of work environment. One participant from the Prevalent group reported, “After I was diagnosed I only worked two more years…it was a combination of taking a rather difficult drug, and a lot of stress…I do think probably had I not started having seizures and taking medication I would have rather worked another 5 years or so”.

### 3.3. Co-Morbidities

Another theme identified was that some participants felt that seizures were less important than their other health problems. One third of the participants from the Incident group (33.3%) felt that that they had other more pressing medical problems to deal with and wished that they did not have to add another problem to their list. Only one participant from the Prevalent group (7.1%) felt the same way. The rest of the participants in both groups recognized epilepsy to be a significant contributor to their decreased quality of life. For one participant in the Prevalent group, seizures were symptoms of another ongoing medical condition. Others had risk factors that contributed to the onset of seizures including alcohol and drug abuse, stroke, dementia, neoplasm, traumatic brain injury, and family history.

### 3.4. Information Gathering

Lack of information about seizures and their management from their healthcare providers was another theme in both groups. This was a source of increased anxiety to the participants and their caregivers. Almost half of the Incident group (45.2%) and all but one participant from the Prevalent group reported that they did not get adequate information about seizures or epilepsy from their healthcare provider. Two individuals from the Incident group did not want information about their seizures. Most of the participants in the Incident group (83.3%) and all the participants in the Prevalent group saw a neurologist for their seizures. One participant in the Incident group noted, “I still don’t have any helpful information (about seizures)…I would like to know what caused it…what to do to prevent another one…how to instruct my husband on what to do if I have one…I feel like I know very little about it”. The participant from the Prevalent group who reported lack of information from the healthcare provider noted, “I was not initially given any information about it, and was told to just take the pills…I did”. Over half (58.3%) of the Incident group and 18.7% of the Prevalent group researched information about epilepsy using resources such as internet, books and pamphlets, as well as seeking information from their healthcare provider.

### 3.5. Stigma

One of the epilepsy related fears and also a contributor to increased disease burden is the fear of being stigmatized by the community. Not surprisingly, those with a longer duration of epilepsy, the Prevalent group (50.0%) believed that the community stigmatizes against those with seizures *versus* less than a quarter of the Incident group (21.4%). Additionally, 57.1% of the Prevalent group and 30.9% of the Incident group also believed that there is a lack of awareness in the community about seizures and epilepsy. One participant from the Incident group noted, “I think it’s under the rug… (it’s not talked about) the same as heart disease or cancer”. Another participant from the Prevalent group noted, “There is a stigma associated with the term epilepsy. Epilepsy has a negative connotation of it being a grand mal seizure and people often don’t know how to respond to those situations”. Prior to the participants experiencing seizures themselves, their own views on seizures were often stereotyped or based on what they were exposed to. One participant reports, “I had a student or two where the nurse warned me that the child was subject to seizures…and (having seizures) were basically negative…cause I don’t know what to do…I was just afraid of it basically, it just seemed so not normal…I just never thought it would happen to me…sort of like any really awful thing”.

### 3.6. Medication Side Effects

Seizure management that included the use of anti-epileptic drug (AED) was another source of decreased quality of life. About half of the participants from both groups (40.4% of the Incident group and 64.2% of the Prevalent group) reported that AED side effects interfered with their daily activities. Most commonly reported AED side effects were fatigue and drowsiness while other side effects mentioned included memory impairment, dizziness and gastrointestinal concerns.

### 3.7. Social Relationships When Living with Seizures/Epilepsy

Participants were asked if their social relationships were affected as a result of seizures. Slightly more than one in five (21.4%) from the Incident group and no one from the Prevalent group reported a change in their relationships with their family members. Lack of friend support was reported by 52.3% of the Incident group compared to only 42.8% of the Prevalent group. Four individuals from the Incident group and one from the Prevalent group chose not to tell people about their diagnosis. One participant from the Incident group said, “I feel that when you have chronic illness, family members and friends have distanced…people don’t want to hear about it…people don’t believe you”. Other participants chose to not tell people because of associated stigma with the diagnosis as mentioned above.

### 3.8. Approach toward Diagnosis

Participants’ personal experience with seizures and their prior exposure to individuals with a seizure disorder or epilepsy earlier on in their life shaped some of their own views on what epilepsy means. When the participants were asked what they thought when they were told that they had a seizure or epilepsy, those from the Prevalent group who were diagnosed earlier in their life (less than 21 years) and who did not have mental problems (e.g., mental retardation) were counseled by their family either to hide the information or the matter was simply not discussed. Those who were diagnosed between 21 and 65 years of age showed diverse reactions, including, refusal to accept diagnosis, disbelief, feeling horrified, terrified, angry, devastated, confused or relieved.

The Incident group had similar reactions although many more felt relief that they knew what they had (although some questioned what it meant to have epilepsy). However, for some in the Incident group, their experiences with seizures combined with other extraneous factors such as the community’s perception of seizures could have contributed to denial of diagnosis (n = 10) and preference for the diagnosis of “seizure disorder” rather than “epilepsy” (n = 6) despite them having an official diagnosis of epilepsy per their medical records. This was not a theme among Prevalent group. One participant from Incident group says, “The word epilepsy has all sorts of negative emotional connotations but seizures not quite that…I will never call it epilepsy but I had a seizure and I take medication for it… I think I have had a seizure is different from saying I have epilepsy”. Of those participants in the Incident group who denied their epilepsy diagnosis, only one experienced generalized tonic-clonic seizures while the rest had more minor seizures. One participant described his symptoms as being fainting spells instead of seizures. He states, “I’ve seen witnessed people that had grand mal seizures and I’ve experienced that as an observer…that would be my idea of what a seizure might be”. About half of the participants from both groups developed a positive attitude in coping with their diagnosis.

## 4. Discussion

The 2010 National health survey showed that adults with epilepsy have a significantly worse health related quality of life in comparison to the general population [5]. In the recent past, other studies have explored the psychosocial impact of epilepsy in older adults. To our knowledge, only a few questionnaire based studies have looked into the effect of age of onset, duration and other seizure-related variables in older adults with epilepsy [3,7,8]. Certain conclusions have been drawn from our interview based qualitative analysis in older adults with epilepsy that are worthy of discussion.

Our study compared quality of life in older adults with newly diagnosed seizures or epilepsy after the age of 65 (Incident group) with older adults with onset of seizures or epilepsy before 65 years (Prevalent group). Consistent with previous studies, quality of life in older adults is affected by the degree of physical and emotional symptoms [3,5,7,8,11,12,13], lack of information about their diagnosis, changes in relationships, having to make significant life changes [13], AEDs [2,5,8,11,13,14,15,16,17] and other co-morbidities [18]. Findings in the present study that were not addressed by Miller and colleagues [13] include the effects of stigma, co-morbidities and the participants’ personal biases and approach to their diagnoses on health related quality of life in older persons with epilepsy.

In our study, no significant difference was seen between the two groups in terms of emotional and physical impact and more than half of both groups reported depression or anxiety. Although the Prevalent group reported slightly higher rates of both physical and emotional symptoms, a larger proportion of the Incident group were receiving depression or anxiety treatment. Decreased levels of physical and emotional functioning are significant contributors to a decreased quality of life [5,12,13]. Previous studies have shown that a later age at onset of epilepsy was associated with an increased risk of mood disorders and anxiety [19,20]. Pugh and colleagues showed initial increased mental and physical limitations in persons with chronic epilepsy compared to those with new onset epilepsy however once their other physical and psychiatric co-morbidities were controlled for, the physical and emotional impact was not significantly different between older adults with chronic epilepsy and those with new onset epilepsy [3]. We are tempted to draw a similar conclusion from our data, but were limited as we did not account for other psychiatric and physical co-morbidities.

In general, the impact of physical limitations including fatigue, drowsiness and cognitive impairment on quality of life was significant in both groups and this effect is compounded by other co-morbidities [3]. A Blessed Information Memory and Concentration (BIMC) test conducted by Haut and colleagues in older adults with epilepsy showed increased cognitive dysfunction in seniors with epilepsy compared to age matched controls without epilepsy [17]. Cognitive limitations as a result of epilepsy in addition to medication side effects are an added source of increased chronic burden in adults with epilepsy [5] and a slightly higher percentage of the Prevalent group reported cognitive limitations which is consistent with a study by Jokeit and colleagues [12] who concluded that cognitive decline was related to increased duration of epilepsy. However, a higher percentage of participants in the Incident group reported that the diagnosis of seizures/epilepsy was viewed as less important than their other health related co-morbidities. This could be because often times in older adults with new onset seizures, the seizures are likely secondary to co-morbidities like cerebrovascular disease, neoplasm and neurodegenerative illnesses that are more debilitating than the seizures themselves [2,15].

Due to the unpredictable nature of seizures, it can be a source of anxiety and worry for the participants and their caregivers. In our study, little difference was seen between the two groups with regards to having to make significant life and lifestyle changes to avoid adverse situations. Laccheo and colleagues reported that worries about seizures were less of a concern in older adults when compared to a younger population [11]. The reasoning behind this conclusion is that the social demands on middle-aged adults can be more complex in terms of the roles they are expected to fulfill during that stage in their life. In our study, significant life changes that the Prevalent group had to make included early retirement and disability, whereas for most of the Incident group it involved having to either move closer to family, into an assisted living facility or have a caregiver. These are all considered significant life changes according to Erickson’s stages of development. In this structure, the primary focus for middle aged adults are work, career and then family. This means that the ability to establish stability within these realms is how one would draw their sense of fulfillment [21]. For older adults, as part of successful aging, the focus is more on the retention their autonomy, physical and mental capacities [4].

Stigma associated with epilepsy is another factor that can have a negative impact on health related quality of life and contribute to the increased disease burden [6]. Significantly more participants in the Prevalent group reported that the community has a lack of awareness about epilepsy and stigmatizes against those with epilepsy. These findings were consistent with that by Baker and colleagues who concluded that the younger population reported more stigma [20]. This along with other factors could explain why only one participant in the Prevalent group was glad to have a diagnosis; however, for many of those in the Incident group, regardless of what the diagnosis was, they were relieved to finally have an explanation for the symptoms they were experiencing. This was also reflected in some participants preferring the term “seizure disorder” to “epilepsy” despite them having an official diagnosis of epilepsy.

Despite the above mentioned seizure related variables that decrease quality of life, the impact of epilepsy decreases with age in comparison to those with new onset epilepsy [3]. Our study showed that a significantly higher percentage of participants in the Prevalent group have developed a positive attitude towards their diagnosis. This might be attributable to the fact that some older adults view past negative experiences more positively over time [3,14]. Lastly as evidenced by our study and prior work by Miller and colleagues [13], a resounding theme has been, the perceived lack of information from healthcare professionals regarding their diagnosis and management. This can be a source of worry and anxiety for the participants and their families. Even though several of the participants used other resources for information, it does bring to light an area that could be improved.

There are limitations to this study. Given the nature of the study, probability of recall bias is high. Also, some of the interviews were provided by the participants’ caregivers and their accounts may not entirely reflect the participants’ perspective. The sample size being smaller in the Prevalent group is another limitation. Since it was an interview based study, determining the exact psychosocial impact of epilepsy especially in the setting of other co-morbidities was not possible.

## 5. Conclusions

This study identified topics that contributed to decreased quality of life in older adults with epilepsy. The results were then compared between seniors with new onset or newly diagnosed seizures after the age of 65 verses seniors with chronic epilepsy to see if the age of onset and duration had any bearing on their quality of life. From the topics that were explored, we conclude that age at onset and duration does seem to be negatively correlated with health related quality of life; however, the perceived health status of older adults with chronic epilepsy was significantly better because they have had a longer opportunity to learn to cope with their diagnosis. The end goal of the study was to provide a richer understanding of the psychosocial impact of seizures in older adults so that healthcare professionals are cognizant of the needs of this specific population. Practical implications for this study includes healthcare providers using this information to not only understand this patient population but also seek out or provide resources in the local community to improve the patient’s quality of life. Clinicians may consider using screening tools for depression and help develop support groups that may enable patients to not only better understand their condition but find comfort in interacting with those who are in a similar situation. This study also identified the need for measures to improve awareness in the local communities about living with epilepsy. A study performed in children was conducted recently by our colleagues in Indiana that highlighted the challenges of diagnosis disclosure in children with their main fears being their desire for normalcy, contending with negative responses to disclosure, in addition to their and others’ perceptions of epilepsy [22]. Expanding on our current research protocol, the authors would like to use this data to conduct a comparison between the adult and pediatric population in the future.
